# Colonization with extended-spectrum beta-lactamase-producing *Escherichia coli* and traveler’s diarrhea attack rates among travelers to India: a systematic review and meta-analysis

**DOI:** 10.1186/s40794-022-00179-1

**Published:** 2022-10-01

**Authors:** Basilua Andre Muzembo, Kei Kitahara, Ayumu Ohno, Keinosuke Okamoto, Shin-Ichi Miyoshi

**Affiliations:** 1 Graduate School of Medicine, Dentistry and Pharmaceutical Sciences, Okayama University, 1-1-1 Tsushimanaka, Kita Ward, Okayama, 700-8530 Japan; 2 Collaborative Research Centre of Okayama University for Infectious Diseases in India, Kolkata 700010, India

**Keywords:** ESBL-EC, Traveler’s diarrhea, International travelers, India, Meta-analysis

## Abstract

**Background:**

India is an attractive destination for travelers. Unfortunately, numerous reports exist on traveler’s diarrhea (TD) and fecal colonization with extended-spectrum beta-lactamase-producing *Escherichia coli* (ESBL-EC) among international travelers visiting India. Here, we systematically reviewed studies published on the acquisition of ESBL-EC and TD attack rates among international visitors to India.

**Methods:**

**Design:** Systematic review and meta-analysis.

A systematic search was performed using Google Scholar, PubMed, EMBASE, Web of Science, and gray literature from 2000 to December 2021, for studies containing data for ESBL-EC acquisition or TD experience related to a trip to India. Random effects models were used to compute the prevalence of ESBL-EC acquisition and TD attack.

**Results:**

The literature search yielded a total of 5023 records. Of these, 31 met our inclusion criteria for systematic review and only 17 could be meta-analyzed (9 for TD, and 8 for ESBL-EC). The overall pooled attack rate of TD was 39% (95% confidence interval, CI: 25–53%). In studies where travelers' memory was used to diagnose TD, the pooled attack rate of TD was slightly higher (42%, 95% CI: 21–64%) compared to those where TD was objectively documented (33%, 95% CI: 17–49%). There were significant risks to be colonized with ESBL-EC among the travelers who experienced TD. The pooled rate of ESBL-EC colonization was 72% (CI: 67–78%). Most ESBL-EC produced CTX-M-15 enzyme. Furthermore, most of the travelers who acquired ESBL-EC were from highly industrialized countries recruited from travel clinics: Canada (*n* = 80), Germany (*n* = 69), Netherlands (*n* = 20), Sweden (*n* = 18), Japan (*n* = 10), Finland (*n* = 8), USA (*n* = 7), Spain (*n* = 5), and Denmark (*n* = 3).

**Conclusions:**

TD pooled attack rate and ESBL-EC acquisition among international travelers visiting India were high in this study. However, we cannot make generalizations based upon this TD pooled attack rate for the current situation, due to a lack of current data. Our study highlights that travelers should be advised on TD to ensure that they do not disregard the risk of contracting TD and be better prepared as a result. It also illustrates the importance of international travel in acquiring antibiotic-resistant *Escherichia coli*.

**Supplementary Information:**

The online version contains supplementary material available at 10.1186/s40794-022-00179-1.

## Introduction

Despite rigorous efforts towards the prevention and reduction of travelers’ diarrhea (TD), it remains one of the most common ailments among travelers worldwide [1]. The illness affects 20–40 million travelers per year depending on the destination, traveler characteristics (such as age), and season of travel [2]. TD is primarily caused by consuming food or water contaminated with microorganisms along with poor hand hygiene [3, 4]. Depending on the country, there are diverse microorganisms that can cause TD. Bacteria are the most common pathogen associated with TD responsible for 80% to 90% of cases (such as enterotoxigenic *Escherichia coli*, enteroaggregative* E*.* coli*, *Campylobacter jejuni*, *Shigella* species, and *Salmonella* species). The next most common causes consist of viruses detected in 5% to 10% of incidences (such as norovirus, rotavirus, and astrovirus), and parasites in 10% of incidences (such as *Giardia lamblia* and *Cryptosporidium*) [5–7]. However, in some cases either the causal agent is unknown or the etiology is revealed as mixed.

The illness is self-limited and short-lived (resolving completely in 1 to 5 days), but it can lead to dehydration [4, 8]. Other sequelae of TD include post-infectious irritable bowel syndrome (PI-IBS), reactive arthritis, and Guillain-Barré syndrome [9, 10]. TD associated with the development of PI-IBS occurs in 3% to 17% of patients [1]. Treatment of TD can have health consequences as well. For instance, treatment of TD with antibiotics while abroad can alter gut microflora and as a consequence can be colonized with extended-spectrum beta-lactamase-producing *E. coli* (ESBL-EC) [11], which is resistant to common antibiotics such as cephalosporins and fluoroquinolones [12]. This event can further contribute to the spread of antimicrobial resistance that may cause downstream infections [13]. Furthermore, TD can also lead to changes in the host microbiome even without antibiotic treatment [14].

In the past, TD attack rates had been reported to be high in India among travelers [15]. In addition, multiple studies have reported fecal colonization with ESBL-EC among international travelers visiting India [16]. One study estimates that annually about one-third of the 100 million travelers to the tropics acquire extended-spectrum beta-lactamase-producing Enterobacteriaceae (ESBL-PE) [17].

Even though there are several published reports on TD and fecal colonization with ESBL-EC related to an international trip to India, no study has systematically synthesized these data. This study aims to summarize the available studies on TD and fecal colonization with ESBL-EC among international travelers to India during the period of 2000 to 2021.

This evidence may help to raise the awareness of both travelers and healthcare professionals about the need for health advisory before travel and educating travelers about preventive measures. We specifically targeted studies carried out in India to understand whether there is any decreasing trend of TD and ESBL-EC acquisition rates as hygiene and improved sanitation has been enhanced in most of the states and union territories (SUTs) by the *Swachh Bharat* (Clean India) Mission.

## Methods

We performed a systematic review and meta-analysis of studies that documented TD and/or fecal colonization with ESBL-EC among international travelers visiting India. In this study, we defined TD as the passage of unformed stools for ≥ 3 times over a span of 24 h with at least one of the following symptoms: nausea; bloody, mucus-containing stool; abdominal cramps; tenesmus; vomiting; fever; or fecal urgency while abroad and/or after returning home [18]. For practical purposes, we considered TD as it was defined in the reviewed studies. This systematic review was undertaken following the Preferred Reporting Items for a Systematic Review and Meta-analysis (PRISMA) guidelines [19] and registered in the international prospective register of systematic reviews (registration number CRD42022324904).

### Data sources and searches

We ran searches on Google Scholar, PubMed, EMBASE, and Web of Science from January 2000 to December 2021. The last search was conducted on 23 December 2021. The following keywords were used for diarrhea: “Traveler’s diarrhea” OR “Traveller’s diarrhoea” OR “Diarrhoea in travellers” OR “Diarrhea in travelers” OR “Traveler with diarrhea” OR “Traveller with diarrhoea”. Search strings also included “extended-spectrum beta-lactamase-producing *Escherichia coli*” OR “extended-spectrum beta-lactamase-producing Enterobacteriaceae”. These keywords were combined with “India” OR “Republic of India”. In addition, we also manually searched the reference lists of the selected studies and related key reviews to retrieve additional records. We also searched for gray literature. We did not use any language restrictions. Potentially relevant studies were exported to Endnote software X9 (Clarivate, Philadelphia, USA) and duplicated references were removed.

### Study selection and data extraction

First, the titles and abstracts of retrieved studies were independently screened by two investigators (BAM and KK). After that, the full texts of potentially relevant studies were retrieved and screened for inclusion. Data were also extracted independently by two investigators (BAM and KK). Disagreements were resolved by consensus. We included studies meeting the following inclusion criteria: (1) a study must have been carried out on international travelers (population); (2) a study must have described individuals with a history of traveling to India (exposure); a study without a mandatory control group (comparison); a study must have described TD and/or fecal colonization with ESBL-EC during their trip in India or after returning back home (outcome); case reports or series published in full text, cross-sectional and prospective cohort studies describing TD and/or fecal colonization with ESBL-EC related to travel to India (study design). Criteria for exclusion from this study included: cases related to the Indian subcontinent (because in this study our target country was India) and those pre-dating 2000 because we wanted to provide the most recent evidence on TD in India. We also excluded review articles, and those on TD in the locals.

For data extraction, we designed a data extraction sheet using Microsoft Excel 2019 (Version 2204, Microsoft Corp., Albuquerque, NM, USA). For each study, extracted information included: first author, year of publication, year of diagnosis/study period, traveler’s country of origin, number of cases, etiology (if reported), performance of stool analysis or only self-reported TD, CTX-M beta-lactamase group, travel duration, area visited, patient demographics (gender and age) wherever applicable. Reported TD risk factors were also extracted. Reporting quality was not assessed because certainty in evidence from case reports and series is usually deemed to be low [20].

### Data synthesis and analysis

Data analysis was performed in Stata version 16 (StataCorp, College Station, TX, USA) using the metaprop command [21]. Descriptive statistics were used to summarize information about the number of cases. The number of cases was grouped by country of origin. For the meta-analysis, the parameters were the number of travelers examined and the proportion of travelers with TD (i.e., TD attack rate) or those with a positive test for ESBL-EC. Studies that did not report the above parameters were excluded from the meta-analysis. Additional analyses were undertaken after stratification by year of diagnosis or study period. Random effects models were used to calculate the pool rates of ESBL-EC acquisition and TD attack. We assessed heterogeneity using I^2^ statistics. I^2^ of > 50% were considered to indicate substantial heterogeneity [22].

## Results

### Literature search and study characteristics

The literature search yielded a total of 5,023 records (Additional file 1), out of which 1,513 duplicates were removed, leaving 3,510 records. We had excluded 3,459 records out of 3,510 after screening their titles and abstracts. Only 51 records were retrieved for full-text screening, of which two were reviews and thus excluded [12, 23]. Another 18 full-texts were excluded mainly due to either a) not being pertinent to this study [13, 24–31], b) referred to the Indian subcontinent [11, 16, 32–35] or c) were carried out prior to 2000 [15, 36, 37]. Finally, 31 records with 859 cases [38–68] met our inclusion criteria for the qualitative synthesis and only 17 of them could be meta-analyzed (9 for TD and 8 for ESBL-EC).

Only one record was published in French [39]; all the others were published in English. They were published between 2005 and 2021, and the study/ diagnostic period was from 2002 to 2019.

The majority of the studies were conducted in travel clinics from high-income countries (Tables 1 and 2). Specifically, the included studies were conducted in the USA [44, 45, 50, 55–57], Canada [66, 67], France [39, 40], Germany [58, 59], India [51, 53], Switzerland [42, 43], Sweden [64, 65], Australia [68], Denmark [62], Finland [54], Japan [61], Korea [38], Netherlands [60], Romania [49], Russia [46], Spain [63], South Africa [48], Thailand [41], and multiple sites (India, UK, and Germany) [47]. Fourteen studies reported on participants who were diagnosed with ESBL-EC after returning from India (Table 2). Of these fourteen studies, ten (71%) clearly reported that ESBL-EC was found to produce CTX-M-15 enzyme [55, 56, 59–61, 63–67].

**Table 1:** Reported cases of traveler’s diarrhea (*n* = 639) in international travelers visiting India and their country of origin

Travelers’ country of origin	Total cases	Number of cases by study	Diagnosis period	Etiology	Disease	Reference: First author (year)	Comments
Korea	8	8	2017	*Salmonella* Typhi H58	Typhoid fever	Shin (2021) [38]	All cases had typhoid fever
France	24	1	2014	*Vibrio cholerae* O1 and *Campylobacter* coli	Cholera	Pougnet (2018) [39]	*Vibrio cholerae *O1, serotype Ogawa
		4	2006	*Vibrio cholerae* O1	Cholera	Tarantola (2008) [40]	-
		19	2006	-	-	Tarantola (2008) [40]	-
Thailand	128	128	2014–2015	NR	-	Olanwijitwong (2017) [41]	Self-reported TD. 86 cases of mild diarrhea, and 42 cases of classic TD. 128 tourists had TD during their stay in India and this number decreased to 19 after returning home
Switzerland	64	30	2013–2014	NR	-	Kuenzli (2017) [42]	Self-reported TD
		34	2013–2014	NR	-	Schindler (2015) [43]	Self-reported TD
USA	51	43	2009–2011	NR	-	Stoney (2017) [44]	Self-reported TD
		8	2007–2010	NR	-	Mackaness (2013) [45]	Self-reported TD
Russia	3	3	2010–2012	*Vibrio cholerae* O1	Cholera	Kuleshov (2016) [46]	Contaminated fruit and drinking fountain water were the potential sources of contamination
UK and Germany	124	124	2009–2010	Enterotoxigenic *Escherichia coli*, Enteroaggregative *E coli*, *Salmonella*, *Aeromonas*, *Entamoeba histolytica*, *Giardia lambia*, and Norovirus	-	Steffen (2013) [47]	This study assessed the efficacy of a patch vaccine against TD. Incidence rate was 18 TD as per primary endpoint in the vaccine group and 18 in the placebo group
South Africa	1	1	2010	*Vibrio cholerae* O1	Cholera	Ismail (2012) [48]	*Vibrio cholerae *O1, serotype Ogawa
Romania	2	2	2009	*Vibrio cholerae* O1	Cholera	Neghina (2012) [49]	*Vibrio cholerae* O1, serotype Ogawa
Australia	1	1	NR	Not identified	-	Zwar (2011) [68]	-
USA and Europe	107	23	2002–2003	Noroviruses and other pathogens	-	Koo (2010) [50]	Study conducted in Goa and Kolkata
		84	2007–2008	Multiple micro-organisms including *Shigella*, Salmonella, *Vibrio*, *Campylobacter* species	-	Jiang (2010) [51]	Study conducted in Goa and Kolkata
UK	31	31	2008	-	-	Tillett (2009) [52]	Sport team during event. Specific preventive measures were taken to prevent diarrhea during the competition. Self-reported TD
Israel, England, Switzerland, Italy, Argentina, South Africa, Austria, USA, Russia, Ireland and Czechia	95	95	2003	-	-	Hillel (2005) [53]	Long-term travelers (median trip duration: 5 months). Self-reported TD

Definition of abbreviations: *TD* Traveler’s diarrhea, *USA* United States of America, *UK* United Kingdom, *NR* Not reported

**Table 2:** Cases of fecal colonization with extended-spectrum beta-lactamase-producing Escherichia coli linked to traveling to India (*n* = 220)

Travelers’ country of origin	Total cases	Number of cases	Study period/diagnosis year	Organisms identified	TD during/after trip	Group of CTX-M beta-lactamase (resistance genes)	Reference: First author (year)	Comment
Finland	8	8	2009–2010	*Escherichia coli*, *Salmonella* and *Campylobacter*	Yes	NR	Kantele (2021) [54]	-
USA	7	1	2017–2019	*Escherichia coli*	Yes	CTX-M-15 and NDM-5	Mellon (2020) [55]	Carbapenemase-producing carbapenem-resistant Enterobacterales was also detected
		2	2009–2010	*Escherichia coli*	-	CTX-M-14 and CTX-M-15	Weisenberg (2012)[56]	-
		4	2011	*Escherichia coli*	-	NR	Islam (2012) [57]	Escherichia coli urinary tract infections in children < 2 years of age
Germany^a^	69	58	2013–2014	*Escherichia coli*	Yes (for some patients)	-	Miranda (2016) [58]	All patients had gastrointestinal complaints including TD. Data on pre-travel ESBL-PE carrier status were not available
		11	2013–2014	*Escherichia coli*	-	CTX-M-15 and CTX-M-27	Lubbert (2015) [59]	Gastroenteritis was a risk factor
Netherlands^a^	20	20	2012–2013	*Escherichia coli*	Yes (for some patients)	CTX-M-15	Reuland (2016) [60]	Not specified whether all cases had TD
Japan	10	10	2011–2012	*Escherichia coli*	Yes	CTX-M-15	Yaita (2014) [61]	-
Denmark	3	3	2011	*Escherichia coli*	-	NR	Lausch (2013) [62]	-
Spain	5	5	2005–2006	*Escherichia coli*	Yes	CTX-M-15	Guiral (2011) [63]	-
Sweden	18	7	2007–2009	*Escherichia coli*	-	CTX-M-15	Tangden (2010) [64]	-
		11	2007–2008	*Escherichia coli*	Yes	CTX-M-15	Tham (2010) [65]	-
Canada	80	14	2004–2006	*Escherichia coli*	-	CTX-M-15	Laupland (2008) [66]	-
		66	2012–2014	*Escherichia coli*	-	CTX-M-15 in 88% (58/66)	Peirano (2017) [67]	Use of antibiotics while in India increased the risk of acquiring ESBL-producing *E. coli*

Definition of abbreviations: *TD* Traveler’s diarrhea, *ESBL* Extended-spectrum beta-lactamase, *USA* United States of America, *NR* Not reported

^a^Not all ESBL-PE strains in these studies were E. coli

The details of records included in this study are available in the supplementary material (Additional file 2). The majority (850/859; 99%) of the travelers with TD and/or ESBL-EC included in this study were diagnosed between 2002 and 2017. In addition, in 41 cases, information on TD was not specifically described [56, 57, 59, 62, 64, 66]. Moreover, TD diagnosis was based solely on traveler’s memory in seven records [41–45, 52, 53] (which may indicate recall bias). In the remaining studies, TD was diagnosed clinically with direct stool examination or with culture [38–40, 46, 48, 49, 51, 68] or polymerase chain reaction (PCR) [47, 50]. ESBL-EC was diagnosed using culture followed by polymerase chain reaction [54, 58, 62, 65] and sequencing [55, 56, 59–61, 63, 64, 66, 67]. In one record, ESBL-EC diagnosis method was not specified [57].

### Pooled attack rate of TD and rate of colonization with ESBL-producing *E. coli*

**Fig. 1:**
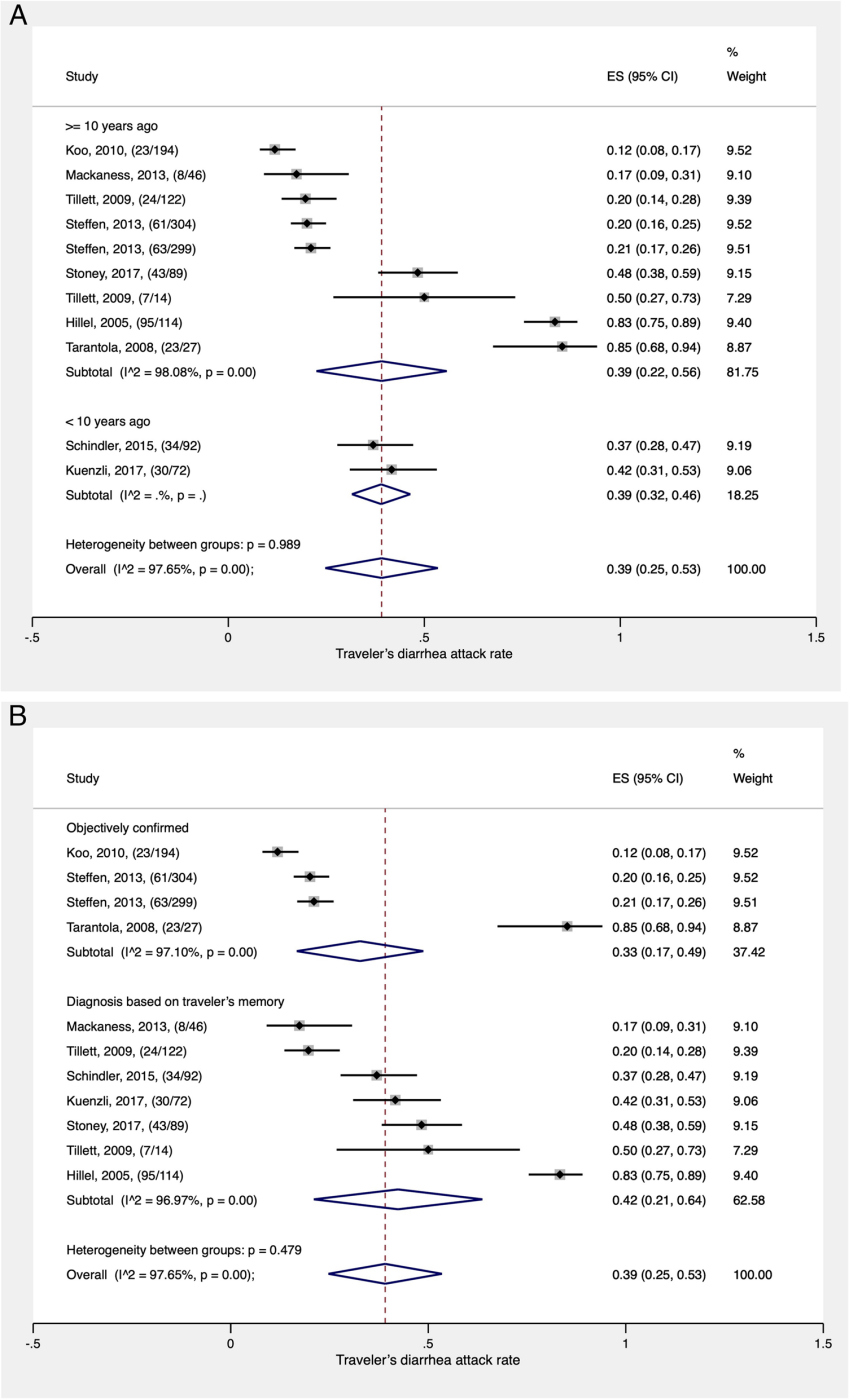
**A** Pooled attack rate of traveler’s diarrhea among international travelers visiting India (Studies are grouped by diagnosis period). Studies are represented by the first author, publication year, and the number of cases/number of travelers examined. The studies by Tillet and Steffen had 2 data points each. ES = effect size; CI = confidence interval. **B** Pooled attack rate of traveler’s diarrhea among international travelers visiting India (Studies are grouped by methods of documenting traveler’s diarrhea). Studies are represented by the first author, publication year, and the number of cases/number of travelers examined. The studies by Tillet and Steffen had 2 data points each. ES = effect size; CI = confidence interval

Nine studies (11 data points), which had sufficient data on TD, were used in the meta-analysis [40, 42–45, 47, 50, 52, 53]. As displayed in Fig. 1A, the overall estimated pooled attack rate of TD was 39% (95% confidence interval, CI: 25–53%). It is important to note that TD was diagnosed between 2002 and 2014 in these studies. Heterogeneity across studies was considerable (I^2^ = 98%) and statistically significant (*p* < 0.001).

In the subgroup analysis, the pooled attack rate of TD varied depending upon the method used to document TD: the pooled attack rate of TD was slightly higher [42% (95% CI:21–64%)] in studies where TD was diagnosed solely on traveler’s memory compared to those where TD was objectively documented [33% (95% CI: 17–49%)] (Fig. 1B).

**Fig. 2:**
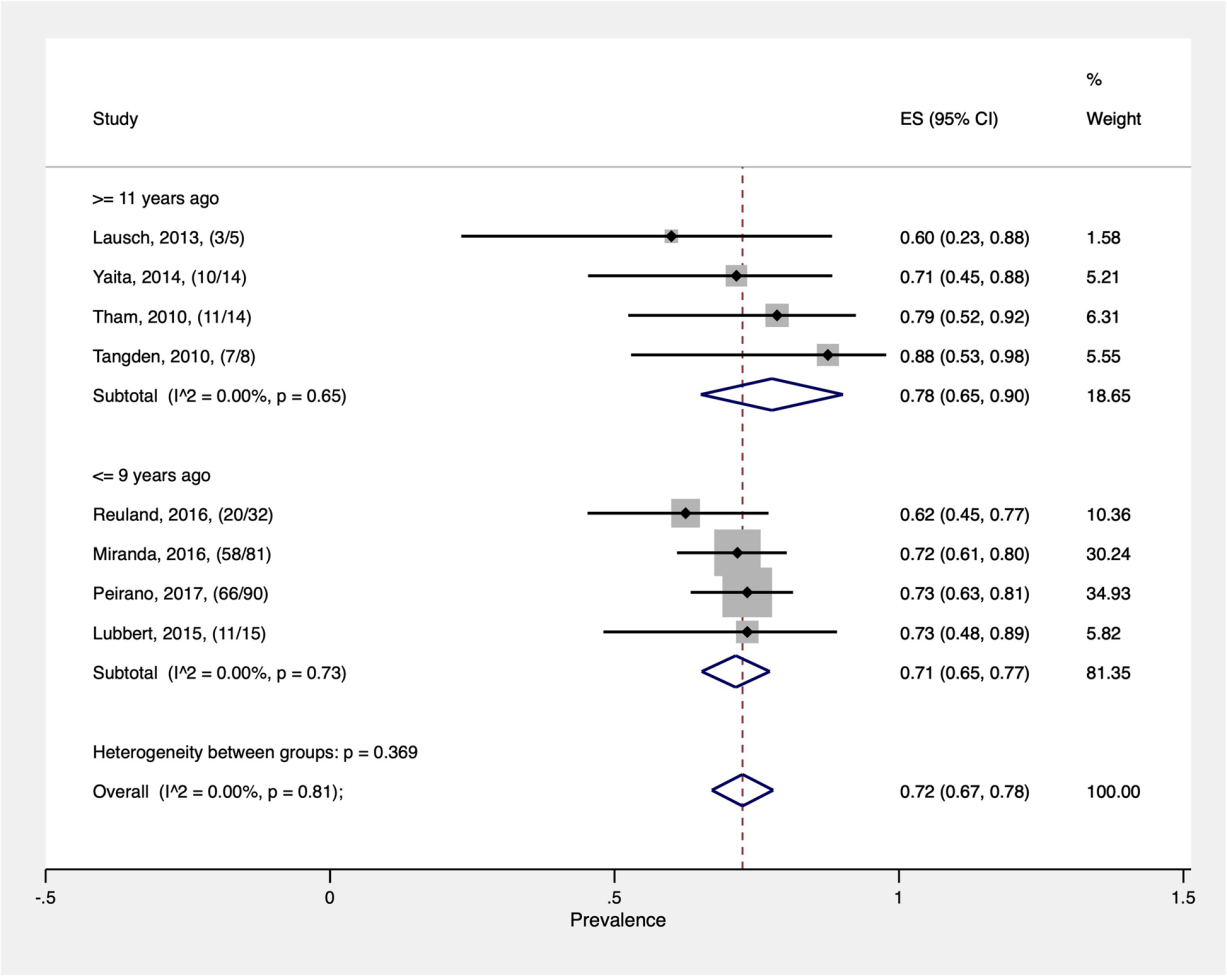
Pooled rate of colonization with extended-spectrum beta-lactamase-producing *Escherichia coli* linked to travel to India. Studies are represented by the first author, publication year, and the number of cases/number of travelers examined. ES = effect size; CI = confidence interval

**Fig. 3:**
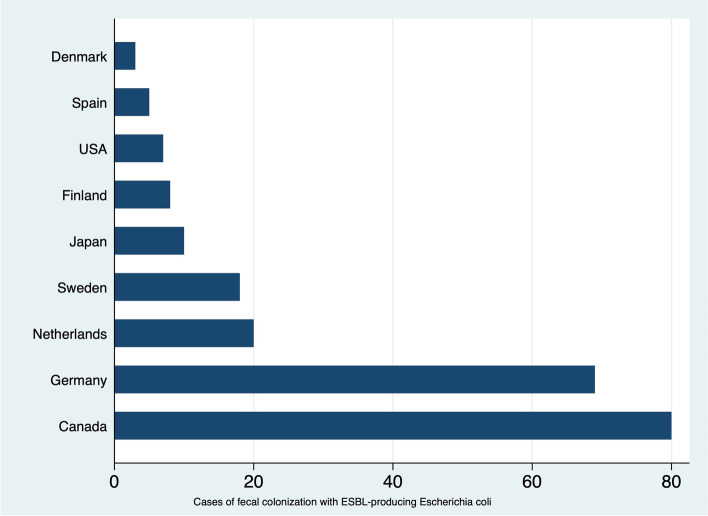
Country of origin of included travelers colonized with extended-spectrum beta-lactamase-producing *Escherichia coli* after visiting India. ESBL = extended-spectrum beta-lactamase

It had been observed that most of the travelers who became colonized with ESBL-EC were more likely to have an history of TD (Table 2). Eight studies were eligible for inclusion in the meta-analysis [58–62, 64, 65, 67]. The overall estimated pooled rate of ESBL-EC acquisition was 72% (CI: 67–78%) (Fig. 2); the rate of ESBL-EC acquisition was quite consistent across the records. Heterogeneity was very low (I^2^ = 0.00%; *p* = 0.81). Subgroup analysis revealed that ESBL-EC acquisition was similar in records where the diagnoses were performed less than 10 years ago [71% (95% CI: 65–77%)] compared with ≥ 11 years ago [78% (95% CI: 65–90%)] (Fig. 2). The traveler's country of origin might also play a role in the acquisition of ESBL-EC after visiting India. Most of the travelers were from highly industrialized countries with Canada (80 cases) and Germany (69 cases) reporting the highest number of cases (Fig. 3).

### Microorganisms causing TD

The etiology of diarrhea was not reported in most of the travelers with self-reported TD [41–45, 52, 53]. In instances where it was documented, however, the causative agents for TD in travelers to India were: *Vibrio cholerae* [39, 40, 46, 48, 49], *Campylobacter *[39, 51, 54], *Salmonella *Typhi [38], noroviruses [50], and multiple other microorganisms (including *Escherichia coli*, *Giardia lamblia*, *shigella* spp. and *Campylobacter* species) [47, 51]. Moreover, in some occasions despite thorough stool examination, the pathogen could not be detected. For instance, there was a negative report for the presence of cholera and other pathogens in the stool sample, even though the traveler showed typical diarrhea symptoms [68]. Co-pathogenicity was also common in cases with ESBL-EC, as seen in one traveler from Finland, who was concomitantly infected with enteropathogenic *E. coli, Salmonella and Campylobacter* [54].

## Discussion

This study summarized the available evidence on TD and fecal colonization with ESBL-EC associated with an international travel to India. We also compared rates of ESBL-EC colonization in studies carried out less than 10 years ago compared with more than 10 years ago after 2002.

The overall pooled attack rate of TD in the reviewed studies reveals that an estimated 39% (around one-third) of the international travelers who visit India may experience TD. This estimate confirms that TD is an important health issue among the international travelers who visit India. To be better prepared, travelers should not disregard the potential risk of contracting TD. Furthermore, it is important to note that this estimate stems from calculations using records where data were collected between 2002 and 2014, which is more than seven years ago. Thus, this estimate might not reflect the current situation as hygienic conditions, economy, and public health programs [69] may have substantially improved in India. Similarly, the provision of safe water and adequate sanitation has been enhanced in most states and union territories by the *Swachh Bharat* (Clean India) Mission [70]. It is also possible that the TD pooled attack rate (39%) may be an underestimate in this study, as either under-reporting of TD is common or because of publication bias. Therefore, it is difficult to extrapolate this estimate to the current situation. Since only a few case reports in this study also provided data collected within the last five years [38, 55], it may be surmised that the COVID-19 pandemic has contributed to the lack of recent data on TD attack rate among international travelers to India.

The pooled attack rate of TD (39%) generated from this study falls within the general range of 30% to 70% as reported by the Centers for Disease Control and Prevention (CDC) [7]. It is also in line with a review by Steffen reporting that in destinations with high TD attack rates such as India, more than 20% of international travelers may experience TD [6]. A similar attack rate of TD (33%) has been reported among international travelers from developed countries visiting Thailand for 28 days [71], among backpackers in Southeast Asia (31%) [72], or again among international climbers in Nepal (36%) [73].

Findings from this review provide evidence that the etiology of TD in visitors to India is heterogenous: bacteria, viruses and parasites, and mixed pathogens have been reported. This suggests that multiple approaches are required for the confirmatory detection of these etiologic agents based on guidelines for TD prevention and treatment [74].

TD will continue to be a challenging health issue for travelers and clinicians. Our findings support the need for clinicians to be aware of the fact that bacteria such as *Vibrio cholerae* can be the cause of TD in travelers who experience acute watery diarrhea after returning from cholera-endemic countries [39, 40, 48, 49]. Hence, we can argue that not seriously considering this possibility may either delay the diagnosis or lead to misdiagnosis. Nevertheless, it is important to note that *Vibrio cholerae* is not a traditional etiology of TD [4, 8, 75].

A key finding of our review shows that the prevalence of ESBL-EC acquisition among visitors to India was 72%. The prevalence was similar between studies performed more than 10 years ago (78%) and less than 10 years ago (71%). This estimated prevalence of 72% is relatively high, but not surprising as ESBL’s endemicity is high in India. To put our observation into context, this may be a common occurrence in India: For instance, Hawser and colleagues have reported high rates of ESBL-EC acquisition among the local population in communities (79%) and hospitals (79%) in India [76]. In comparison with the data from other Asian countries, the rates of ESBL-EC acquisition in local communities vary between 51 to 71% in Southeast Asia (such as Vietnam, Thailand, and Laos) [77].

The ESBL-EC prevalence of 72% found in this study among visitors to India closely mirrors that reported in visitors to Egypt (71%) [78]. Moreover, one study found an ESBL-PE prevalence of 75% among Dutch travelers returning from southern Asia [16].

Our findings provide some evidence that international travel contributes to the acquisition of antimicrobial-resistant ESBL-EC. This has implications in public health because antimicrobial resistance may cause downstream infections [13, 79]. We should also note that some authors consider that because ESBL’s endemicity is wider, the contribution of international travel towards the spread of ESBL is relatively minor when compared with endemicity, which is much wider [80]. Most of the travelers with ESBL-EC colonization developed TD (a frequently reported risk factor of acquiring ESBL-EC). In general, for the international travelers, there are various risk factors for becoming colonized by multiresistant bacteria including ESBLs. These risk factors include travel destination, antibiotic use [11, 81] and TD [82]. Thus, these risk factors serve as a reminder for the judicious use of antibiotics, and suggests that there is a need to improve practices in antibiotic use when treating TD.

ESBL-EC colonization is usually transient in returning travelers but can persist for a year before the patient’s system fully recovers. Before clearance, ESBL-EC can be transmitted to other household members, with a probability of transmission rate at 12% [16]. This is plausible and thus a serious problem because most ESBL producing Enterobacteriaceae can lead to difficult-to-treat infections due to multi-drug resistance.

Although this study provides valuable insights into planning future studies in order to help understand TD and ESBL-EC acquisition among visitors to India, it has several limitations. First, our review was restricted to studies carried out in India. Our approach of only taking into account studies that separately provide rates for India excludes large studies reporting on ESBL-PE rates among travelers to South Asia. Therefore, we are now working on another meta-analysis study in order to reflect a true representation of the Indian subcontinent including only prospective studies.

Second, despite our wide search strategy, we could have missed some records and case reports. Case reports do not report all TD cases or ESBL colonization, and this study may also be vulnerable to publication bias. Lack of data precluded the quantitative analysis of travelers’ behaviors associated with ESBL-EC colonization. Behaviors such as antimicrobials consumption (especially fluoroquinolones) during travel increase the risk of ESBL-PE acquisition [12].

Third, because of caveats in the data stratified by variables, such as the region of India visited (rural or urban) in primary studies, destinations within India were not considered in the sensitivity analysis. These factors may need to be considered in future studies.

## Conclusions

In conclusion, TD pooled attack rate and ESBL-EC acquisition among international travelers who experienced TD after visiting India were found to be high in this study. The etiology of TD was heterogeneous which includes a wide variety of bacteria, viruses, parasites, and mixed pathogens and therefore, their detection requires different types of methods. Summarized data provide practical implications in travel medicine. Firstly, our study highlights that travelers should be advised on TD to ensure that they do not disregard the potential risk of contracting TD and, as a result, be better prepared. Thus, pre-travel counseling is justified. Secondly, there is a need to remind health care practitioners that in the case of a returning traveler with multidrug resistant *Escherichia coli*, some antimicrobials such as cephalosporins and fluoroquinolones may be ineffective and therefore precautions need to be taken.

## Supplementary Information

**Additional file 1: Appendix Figure 1.** PRISMA diagram summarizing evidence search and study selection.

**Additional file 2: Supplementary Table 1.** Characteristics of reviewed records.

## Data Availability

All relevant data are within the manuscript and its supporting information files.

## Abbreviations

CDC: Centers for Disease Control and Prevention
COVID 19: Coronavirus disease 2019
EC: *Escherichia coli*
ESBL-EC: Extended-spectrum beta-lactamase-producing *Escherichia coli*
ESBL-PE: Extended-spectrum beta-lactamase-producing Enterobacteriaceae
PI-IBS: Post-infectious irritable bowel syndrome
PRISMA: Preferred Reporting Items for a Systematic Review and Meta-analysis
TD: Traveler’s diarrhea
SUTs: States and union territories
