# Supplementary material for: Colonization with extended-spectrum beta-lactamase-producing Escherichia coli and traveler’s diarrhea attack rates among travelers to India: a systematic review and meta-analysis
Source: Trop Dis Travel Med Vaccines. 2022 Oct 1;8:22. doi: 10.1186/s40794-022-00179-1 (PMC9525155; doi:10.1186/s40794-022-00179-1)
Supplement: Supplementary file 1 — Additional file 1: Appendix Figure 1. PRISMA diagram summarizing evidence search and study selection. [file 40794_2022_179_MOESM1_ESM.docx]

**Identification of studies via grey literature and hand search**

**Identification of studies via databases**

Records identified from:

Websites (n = 1)

Citation searching (n = 6)

Records removed *before screening*:

Duplicate records removed (n = 1513)

Records identified from:

Databases (n = 5016)

PubMed: 841

EMBASE: 484

Web of Science:115

Google Scholar: 3576

**Identification**

Records screened

(n = 3503)

Records excluded

(n = 3459)

Reports not retrieved

(n = 0)

Reports sought for retrieval

(n = 7)

Reports sought for retrieval

(n = 44)

Reports not retrieved

(n = 0)

**Screening**

Reports assessed for eligibility

(n = 7)

Reports excluded (n = 6):

Not pertinent to this study (n = 4);

Records on the Indian subcontinent (n = 2)

Reports assessed for eligibility

(n = 44)

Reports excluded (n = 14):

Review (n = 2);

Not pertinent to this study (n = 5);

Records on the Indian; subcontinent (n = 4);

Diagnosis pre-dating 2000 (n = 3)

Studies included in review

(n = 31)

**Included**

***Source of template****:*  Page MJ, McKenzie JE, Bossuyt PM, Boutron I, Hoffmann TC, Mulrow CD, et al. The PRISMA 2020 statement: an updated guideline for reporting systematic reviews. BMJ 2021;372:n71. doi: <https://doi.org/10.1136/bmj.n71>
