# Supplementary material for: Colonization with extended-spectrum beta-lactamase-producing Escherichia coli and traveler’s diarrhea attack rates among travelers to India: a systematic review and meta-analysis
Source: Trop Dis Travel Med Vaccines. 2022 Oct 1;8:22. doi: 10.1186/s40794-022-00179-1 (PMC9525155; doi:10.1186/s40794-022-00179-1)
Supplement: Supplementary file 2 — Additional file 2: Supplementary Table 1. Characteristics of reviewed records. [file 40794_2022_179_MOESM2_ESM.docx]

**Supplementary Table 1. Characteristics of reviewed records**

| **Study first author (publication year)** | **Study design** | **Study participant nationality** | **Disease** | **Year (s) of diagnosis** | **Number of cases, n (%)** | **Sample size** | **Age (years); gender** | **Traveler type** | **Travel duration (days)** | **Travelers ill during travel (yes or no)** | **Travelers ill after travel (yes or no)** | **Etiology** | **Visited region** | **Comments** |
| --- | --- | --- | --- | --- | --- | --- | --- | --- | --- | --- | --- | --- | --- | --- |
| Shin (2021)^1^ | Case series | Korea | TD (Typhoid fever) | 2017 | 8 | 8 | NR | Tourist | NR |  | yes | Salmonella Typhi H58 | Northwest (New Delhi, Amritsar, Dharamshala, and Agra) |  |
| Kantele (2021)^2^ | Prospective cohort | Finland | TD (ESBL-PE) | 2009-2010 | 8 |  | 31; female |  | 11 |  |  | EPEC |  | ESBL-DEC |
|  | | | | |  |  | 56; female |  | 7 |  |  | EAEC |  |  |
|  |  |  |  |  |  |  | 47; male |  | 16 |  |  | EAEC |  |  |
|  |  |  |  |  |  |  | female; 22 |  | 14 |  |  | ETEC |  |  |
|  |  |  |  |  |  |  | 20; male |  | 16 |  |  | EAEC, Salmonella and Campylobacter |  |  |
|  |  |  |  |  |  |  | 31; male |  | 27 |  |  | EAEC and Campylobacter |  |  |
|  |  |  |  |  |  |  | 25; male |  | 32 |  |  | EAEC |  |  |
|  |  |  |  |  |  |  | 59; male |  | 13 |  |  | EAEC |  |  |
| Mellon (2020)^3^ | Case report | USA | TD (ESBL) | 2017-2019 | 1 |  | 48; female |  | 9 | yes |  | Escherichia coli |  | Carbapenemase-producing carbapenem-resistant Enterobacterales and Beta-lactamases CTX-M-15 were detected |
| Pougnet (2018)^4^ | Case report | France | TD (Cholera) | 2014 | 1 |  | 54; male |  |  |  |  | Vibrio cholerae O1 (Ogawa) and campylobacter coli |  |  |
| Olanwijitwong (2017)^5^ | Cross-sectional | Thailand | TD | 2014-2015 | 128 (86 cases of mild diarrhea, and 42 cases of classic TD) |  |  | Tourist | 11 | yes | yes |  |  | Self-reported TD |
| Kuenzli (2017)^6^ | Cross-sectional | Switzerland | TD | 2013-2014 | 30 | 72 |  | Tourist |  | yes | yes |  |  | Self-reported TD |
| Stoney (2017)^7^ | Cross-sectional | USA | TD | 2009-2011 | 43 | 89 |  | Tourist |  | yes | yes |  |  | Self-reported TD |
| Peirano (2017)^8^ | Prospective cohort | Canada | ESBL | 2012-2014 | 66 | 90 |  |  |  |  |  | Escherichia coli |  |  |
| Miranda (2016)^9^ | Prospective cohort | Germany | Gastrointestinal complaints including TD (ESBL-PE) | 2013-2014 | 58 | 81 |  |  |  |  |  | Escherichia coli |  | All patients had gastrointestinal complaints. CTX-M group in most of the cases. No data on pre-travel ESBL-PE carrier status |
| Reuland (2016)^10^ | Prospective cohort | Netherlands | TD (ESBL-PE): Not specified whether all cases had TD | 2012-2013 | 20 | 32 |  |  |  |  |  | Escherichia coli |  | CTX-M-15 was dominant |
| Kuleshov (2016)^11^ | Cross-sectional | Russia | TD (Cholera) | 2012 |  |  | 28; male |  |  |  |  | Vibrio cholerae O1 | Srinagar | Probable source of contamination: Drinking fountain water or playing in river |
|  |  |  |  | 2010 |  |  | 25; female |  |  |  |  | Vibrio cholerae O1 |  | Probable source of contamination: Contaminated fruit |
|  |  |  |  | 2010 |  |  | 29; female |  |  |  |  | Vibrio cholerae O1 |  | Probable source of contamination: Fruit rinsed in tap water |
| Schindler (2015)^12^ | Prospective cohort | Switzerland | TD | 2013-2014 | 34 | 92 |  | Tourist |  |  |  |  |  | Self-reported TD |
| Lubbert (2015)^13^ | Prospective cohort | Germany | - | 2013-2014 | 11 | 15 |  |  |  |  |  | Escherichia coli |  | Beta-lactamases: CTX-M-15 and CTX-M-27. Gastroenteritis was a risk factor. Extended-spectrum beta-lactamase-producing Enterobacteriaceae |
| Yaita (2014)^14^ | Case-control | Japanese | TD | 2011-2012 | 10 | 14 |  |  |  |  |  | Escherichia coli |  | TD in all cases. Extended-spectrum beta-lactamase-producing Enterobacteriaceae |
| Steffen (2013)^15^ | Randomized controlled trial | UK and Germany | TD | 2009-2010 | 63 | 299 | 32 |  | 17 |  |  | ETEC, EAEC, Salmonella, Aeromonas, Entamoeba histolytica, Giardia lambia, and Norovirus | Goa, Delhi, Varanasi, Kolkata | Incidence rate was 18 TD as per primary endpoint in the vaccine group |
|  |  |  | TD | 2009-2010 | 61 | 304 | 32 |  | 17 |  |  |  |  | Incidence rate was 18 TD as per primary endpoint in the placebo group |
| Lausch (2013)^16^ | Cross-sectional | Denmark | - | 2011 | 3 | 5 |  |  |  |  |  | Escherichia coli |  | Extended-spectrum beta-lactamase-producing Enterobacteriaceae |
| Mackaness (2013)^17^ | Retrospective observational | USA | TD | 2007-2010 | 8 | 46 |  |  |  |  |  |  |  | Self-reported TD |
| Weisenberg (2012)^18^ | Cross-sectional | USA | - | 2009-2010 | 2 |  |  |  |  |  |  | Escherichia coli |  | Extended-spectrum beta-lactamase-producing Enterobacteriaceae. Beta-lactamases: CTX-M-14 and CTX-M-15 genes |
| Islam (2012)^19^ | Case report | USA | - | 2011 | 4 |  | < 2 |  |  |  |  | Escherichia coli |  | Extended-spectrum beta-lactamase-producing Escherichia coli. Escherichia coli urinary tract infections in children < 2 years of age |
| Ismail (2012)^20^ | Case report | South Africa | TD (cholera) | 2010 | 1 |  | 37; female |  |  |  |  | Vibrio cholerae O1 |  | Vibrio cholerae O1, serotype Ogawa |
| Neghina (2012)^21^ | Case report | Romania | TD (cholera) | 2009 | 1 |  | 53, female |  | 14 |  |  | Vibrio cholerae O1 | Northern India (Himachal Pradesh) | Vibrio cholerae O1, serotype Ogawa |
|  |  |  | TD |  | 1 |  |  |  |  |  |  |  |  |  |
| Guiral (2011)^22^ | Case report | Spain | TD | 2005-2006 | 5 |  |  |  |  |  |  | Escherichia coli |  | Extended-spectrum beta-lactamase-producing Escherichia coli. Beta-lactamases: CTX-M-15 |
| Zwar (2011)^23^ | Case report | Australia | TD | - |  |  | 21, female |  |  |  |  | None identified |  |  |
| Koo (2010)^24^ | Prospective cohort | USA and Europe | TD | 2002-2003 | 23 | 194 |  |  |  |  |  | Noroviruses |  | Goa and Kolkata |
| Jiang (2010)^25^ |  | USA and Europe | TD | 2007-2008 | 48 |  |  |  |  |  |  | Mixed pathogens including shigella, salmonella, vibrio, campylobacter, arcobacter butzleri, enterotoxigenic bacteroides fragilis, and enterotoxigenic Escherichia coli |  | Goa |
|  |  |  | TD | 2008 | 36 |  |  |  |  |  |  | Shigella, campylobacter, arcobacter butzleri, enterotoxigenic bacteroides fragilis, and ETEC |  | Kolkata |
| Tangden (2010)^26^ | Prospective cohort | Sweden | - | 2007-2009 | 7 | 8 |  |  |  |  |  | Escherichia coli |  | Extended-spectrum beta-lactamase-producing Escherichia coli. Beta-lactamases: CTX-M-15 |
| Tham (2010)^27^ | Cross-sectional | Sweden | TD | 2007-2008 | 11 | 14 |  |  |  |  |  | Escherichia coli |  | Extended-spectrum beta-lactamase-producing Escherichia coli. Beta-lactamases: CTX-M-15 |
| Tillett (2009)^28^ | Cross-sectional | UK | TD | 2008 | 24 | 122 |  | Sport team (during event) | 9 | yes |  |  | Pune | 20 athletes and 4 officials had TD (Specific preventive measures were taken to prevent diarrhea). Self-reported TD |
|  |  |  | TD | 2008 | 7 | 14 |  | Sport team (during the reconnaissance trip) |  |  |  |  |  | No specific preventive measures were taken during the reconnaissance trip |
| Laupland (2008)^29^ | Prospective cohort | Canada | - | 2004-2006 | 14 |  |  |  |  |  |  | Escherichia coli |  | Extended-spectrum beta-lactamase-producing Escherichia coli. Beta-lactamases: CTX-M-15 |
| Tarantola (2008)^30^ | Case report | France | TD | 2006 | 23 | 27 |  |  | 9 |  |  |  |  |  |
|  |  |  | TD (Cholera) | 2006 | 4 | 23 |  |  | 9 |  |  | Vibrio cholerae O1 |  |  |
| Hillel (2005)^31^ |  | Israel, UK, Switzerland, Italy, Argentina, South Africa, Austria, USA, Russia, Ireland and Czechia | TD | 2003 | 95 | 114 | 20-50 |  |  | yes |  |  |  | Long-term travelers (median trip duration: 5 months) |

Definition of abbreviations: TD= traveler’s diarrhea; NR= not reported; ESBL-DEC= extended-spectrum beta-lactamase-producing diarrhoeagenic Escherichia coli; ESBL-PE= extended-spectrum beta-lactamase-producing enterobacteriaceae; EAEC= enteroaggregative Escherichia coli; EPEC= enteropathogenic Escherichia coli; ETEC= enterotoxigenic Escherichia coli; USA= United States of America; UK= United Kingdom.

**References**

1 E Shin, J Park, HJ Jeong, et al. Emerging high-level ciprofloxacin-resistant salmonella enterica serovar typhi haplotype h58 in travelers

returning to the republic of korea from india. PLoS Negl Trop Dis 2021; 15(3):e0009170.

2 A Kantele, T Laaveri. Extended-spectrum beta-lactamase-producing strains among diarrheagenic escherichia coli-prospective traveler study

with literature review. J Travel Med 2021; 17; 29(1):taab042.

3 G Mellon, SE Turbett, C Worby, et al. Acquisition of antibiotic-resistant bacteria by u.S. International travelers. N Engl J Med 2020;

382(14):1372-1374.

4 L Pougnet, R Pougnet, A Voarino, et al. [cholera in brest, france]. Ann Biol Clin (Paris) 2018; 76(1):107-110.

5 J Olanwijitwong, W Piyaphanee, K Poovorawan, et al. Health problems among thai tourists returning from india. J Travel Med 2017; 24(4).

6 E Kuenzli, D Juergensen, K Kling, et al. Previous exposure in a high-risk area for travellers' diarrhoea within the past year is associated with a

significant protective effect for travellers' diarrhoea: A prospective observational cohort study in travellers to south asia. J Travel Med 2017;

24(5).

7 RJ Stoney, PV Han, ED Barnett, et al. Travelers' diarrhea and other gastrointestinal symptoms among boston-area international travelers.

American Journal of Tropical Medicine and Hygiene 2017; 96(6):1388-1393.

8 G Peirano, DB Gregson, S Kuhn, et al. Rates of colonization with extended-spectrum beta-lactamase-producing escherichia coli in canadian

travellers returning from south asia: A cross-sectional assessment. CMAJ Open 2017; 5(4):E850-E855.

9 IB Miranda, R Ignatius, R Pfuller, et al. High carriage rate of esbl-producing enterobacteriaceae at presentation and follow-up among travellers

with gastrointestinal complaints returning from india and southeast asia. J Travel Med 2016; 23(2).

10 EA Reuland, GJB Sonder, I Stolte, et al. Travel to asia and traveller's diarrhoea with antibiotic treatment are independent risk factors for

acquiring ciprofloxacin-resistant and extended spectrum beta-lactamase-producing enterobacteriaceae-a prospective cohort study. Clin

Microbiol Infec 2016; 22(8).

11 KV Kuleshov, SO Vodop'ianov, VG Dedkov, et al. Travel-associated vibrio cholerae o1 el tor, russia. Emerg Infect Dis 2016; 22(11):2006-

2008.

12 VM Schindler, VK Jaeger, L Held, et al. Travel style is a major risk factor for diarrhoea in india: A prospective cohort study. Clin Microbiol

Infect 2015; 21(7):676 e1-4.

13 C Lubbert, L Straube, C Stein, et al. Colonization with extended-spectrum beta-lactamase-producing and carbapenemase-producing

enterobacteriaceae in international travelers returning to germany. Int J Med Microbiol 2015; 305(1):148-56.

14 K Yaita, K Aoki, T Suzuki, et al. Epidemiology of extended-spectrum beta-lactamase producing escherichia coli in the stools of returning

japanese travelers, and the risk factors for colonization. PLoS One 2014; 9(5):e98000.

15 R Steffen, JP Cramer, G Burchard, et al. Efficacy of a travelers' diarrhea vaccine system in travelers to india. J Travel Med 2013; 20(6):374-9.

16 KR Lausch, K Fuursted, CS Larsen, M Storgaard. Colonisation with multi-resistant enterobacteriaceae in hospitalised danish patients with a

history of recent travel: A cross-sectional study. Travel Med Infect Di 2013; 11(5):320-323.

17 CA Mackaness, A Osborne, D Verma, et al. A quality improvement initiative using a novel travel survey to promote patient-centered

counseling. J Travel Med 2013; 20(4):237-42.

18 SA Weisenberg, JR Mediavilla, L Chen, et al. Extended spectrum beta-lactamase-producing enterobacteriaceae in international travelers and

non-travelers in new york city. PLoS One 2012; 7(9):e45141.

19 S Islam. High rates of esbl-producing escherichia coli in young children who traveled recently to india. Pediatr Infect Dis J 2012; 31(10):1103-

1103.

20 H Ismail, AM Smith, BN Archer, et al. Case of imported vibrio cholerae o1 from india to south africa. J Infect Dev Ctries 2012; 6(12):897-

900.

21 R Neghina, AM Neghina. A case of imported cholera in romania in 2009. Infect Dis Clin Pract 2012; 20(148-149.

22 E Guiral, E Mendez-Arancibia, SM Soto, et al. Ctx-m-15-producing enteroaggregative escherichia coli as cause of travelers' diarrhea. Emerg

Infect Dis 2011; 17(10):1950-3.

23 NA Zwar, A Torda. Investigation of diarrhoea in a traveller just returned from india. BMJ 2011; 342(d2978.

24 HL Koo, NJ Ajami, ZD Jiang, et al. Noroviruses as a cause of diarrhea in travelers to guatemala, india, and mexico. J Clin Microbiol 2010;

48(5):1673-6.

25 ZD Jiang, HL Dupont, EL Brown, et al. Microbial etiology of travelers' diarrhea in mexico, guatemala, and india: Importance of

enterotoxigenic bacteroides fragilis and arcobacter species. J Clin Microbiol 2010; 48(4):1417-9.

26 T Tangden, O Cars, A Melhus, E Lowdin. Foreign travel is a major risk factor for colonization with escherichia coli producing ctx-m-type

extended-spectrum beta-lactamases: A prospective study with swedish volunteers. Antimicrob Agents Chemother 2010; 54(9):3564-8.

27 J Tham, I Odenholt, M Walder, et al. Extended-spectrum beta-lactamase-producing escherichia coli in patients with travellers' diarrhoea.

Scand J Infect Dis 2010; 42(4):275-280.

28 E Tillett, M Loosemore. Setting standards for the prevention and management of travellers' diarrhoea in elite athletes: An audit of one team

during the youth commonwealth games in india. Brit J Sport Med 2009; 43(13):1045-1048.

29 KB Laupland, DL Church, J Vidakovich, et al. Community-onset extended-spectrum beta-lactamase (esbl) producing escherichia coli:

Importance of international travel. J Infect 2008; 57(6):441-8.

30 A Tarantola, J Vaucel, C Laviolle, et al. A cluster of vibrio cholerae o1 infections in french travelers to rajasthan (india), may 2006. J Travel

Med 2008; 15(4):273-277.

31 O Hillel, I Potasman. Correlation between adherence to precautions issued by the who and diarrhea among long-term travelers to india. J

Travel Med 2005; 12(5):243-7.
